# Adhesion of Self-Complementary, Sinusoidal Surfaces Fabricated Using Two-Photon Polymerization

**DOI:** 10.1021/acsapm.5c02773

**Published:** 2025-09-25

**Authors:** Madelyn P. Jeske, Hannan Wang, Hesam Askari, David R. Harding, Mitchell Anthamatten

**Affiliations:** † Department of Chemical Engineering, 6927 University of Rochester, 4306 Wegmans Hall, Rochester, New York 14627, United States; ‡ Laboratory for Laser Energetics, University of Rochester, 250 East River Road, Rochester, New York 14623, United States; § Department of Mechanical Engineering, University of Rochester, 235 Hopeman Bldg., Rochester, New York 14627, United States

**Keywords:** switchable adhesion, two-photon polymerization, self-complementary surfaces, shape-memory polymers, polymer networks

## Abstract

Microscale, pick-and-place assembly is a non-lithographic assembly method poised to impact diverse fields including flexible electronics, microfluidics and robotics. However, a major technological challenge is the need to deterministically control adhesion between parts. Here, switchable adhesion involving 3D-printed, self-complementary surfaces is demonstrated. Mechanical properties of metasurfaces pressed against flat, rigid substrates are modeled using finite element methods. A series of flat slabs and metastructured slabs with 2D sinusoidal surfaces are printed using two-photon polymerization (2PP) of a shape-memory resin. The surface frequency of featured slabs was varied between 3.3̅ mm^–1^ and 26.6̅ mm^–1^ with similar amplitudes. Adhesion between printed metasurfaces and glass and between printed, self-complementary metasurfaces is studied above and below the cured resin’s glass transition temperature (∼45 °C). Simple heating of adhering surfaces to above 60 °C lowers adhesion, and compression of surfaces while above the glass transition temperature followed by cooling to room temperature elevates adhesion. The nominal adhesive strength between printed, self-complementary surfaces, as determined by the maximum observable pull-off stress, exceeds 3 MPa. Further tailoring complementary surfaces for adhesion control may facilitate microscale disassembly for recovery of components or precious metals.

## Graphical abstract

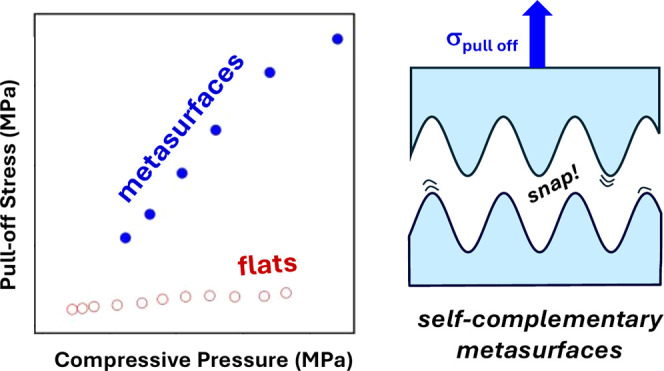

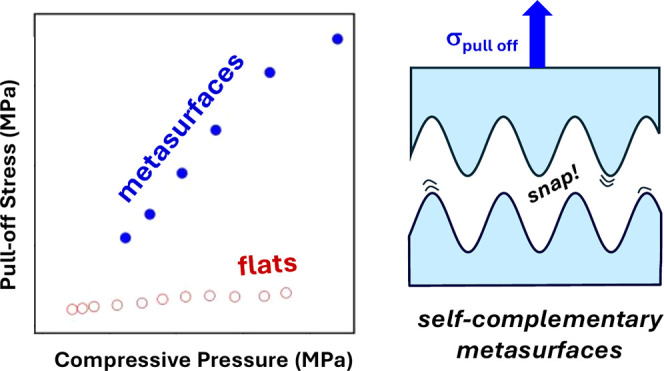

## Introduction

Non-lithographic, microscale assembly methods have emerged to meet specialized manufacturing demands in fields such as flexible electronics, photonics, microfluidics, and robotics. Conventional microassembly involves deterministic pickup and placement of microscopic parts where adhesion control is paramount. This requires prefabricated parts to be manipulated using micromanipulators, grippers, or vacuum chucks; and femtoliter (fL) or picoliter (pL) quantities of glue are used to adhere parts together.^1^ Physical interactions such as electrostatic or van der Waals interactions can also be used to hold parts in place, obviating the use of glue.^2–4^ Although conventional microassembly is time-consuming and is prone to assembly error, the method is routinely used for specialized fabrication, e.g. the assembly, mounting and robotic handling of targets for high power laser facilities.^5,6^ Approaches to controlling adhesion for microassembly are diverse and have included subsurface stiffness or pressure modulation,^7–10^ magnetically actuatable patterns,^11^ and thermally activated liquid crystalline stress changes.^12^ Adhesion control is equally important in microcontact printing (i.e., soft lithography) which is an additive manufacturing method to pattern-transfer an ink to a target substrate via an elastomeric stamp. A variety of inks can be patterned including organic molecules, polymer layers, colloid particles or even cells. During ink pickup, the ink should more strongly adhere to the stamp, and during ink transfer, the ink should more strongly adhere to the target substrate. Methods to modulate the adhesion during elastomeric printing have included kinetically control stamping,^13,14^ shape-memory triggered pickup and delamination,^15^ and heat-triggered air pressure changes within microcavities.^16^

Inspired by the remarkable adhesion abilities of geckos, octopuses, and snails, researchers have developed microstructured, soft surfaces with varying levels of dry and wet adhesion.^17–22^ The level of adhesive strength depends on feature shape, material stiffness, and interfacial surface properties. The underlying physical principle is that low-energy, elastic deformation of protruding surface features enables more conformal contact, permitting short-range interactions, such as van der Waals forces, to contribute to adhesion. Furthermore, the features’ shape and stiffness affect interfacial crack formation and propagation under tension or shear, limiting the maximum achievable adhesion.^23^ Advances in high resolution 3D printing have enabled a variety of surface motifs to be prepared including pillars, fibers, and mushrooms with each geometry offering advantages and limitations to adhesion control.^22,24,25^ While the majority of soft-adhesion studies have focused on the interaction between a microstructured surface and a flat surface, the interaction between soft, self-complementary surfaces that perfectly fit together can make full use of all available surface area.^26–29^ Further, self-complementary surfaces can be “keyed” for shape recognition or self-aligning. For example, ridges or fibrils can interdigitate to realize enhanced surface contact when two surface are oriented parallel to each other.^29^

Here we apply two-photon polymerization (2PP) to fabricate shape-memory slabs with 2D sinusoidal surfaces, and we study the contact mechanics between printed flat surfaces; printed surfaces and glass substrates; and between self-complementary pairs of printed surfaces. Unlike fibers or high aspect ratio pillars that interact laterally by clustering or matting,^30^ sinusoidal features are mechanically isolated from one another, and the 2D spatial frequency can be systematically varied to provide different contact surface areas. We formulate resins for selective thiol-Micheal coupling; similarly formulated resin systems were shown capable of printing highly uniform shape-memory polymer networks at high resolution.^31,32^ Cured resins exhibit a glass-to-elastomer transition temperature around 45 °C, enabling the printed material to transition from a stiff, glassy state to a soft, elastomeric state upon heating, with a corresponding drop in Young’s modulus by more than 2 orders of magnitude. By varying the spatial frequencies of the sinusoidal surfaces, the amount of contact area available for adhesion is systematically increased. Further, we present evidence that adhesion can be switched on by cooling while under compression, and adhesion can be reduced simply by heating.

## Materials and Methods

### Resin Preparation

Two-photon polymerization (2PP) resins were formulated by mixing pentaerythritol tetrakis (3-mercaptopropionate) (PETMP) and 1,3,5-triallyl-1,3,5-triazine-2,4,6­(1*H*, 3*H*, 5*H*)-trione (TTT) in a 1:1 molar ratio of thiol to allyl functional groups, corresponding to a stoichiometric balance for step-growth polymerization. To this mixture, 2 wt % of 9-anthrylmethyl *N*,*N*-diethylcarbamate was added as a photobase generator, 2 wt % of isopropylthioxanthone (ITX) as a photosensitizer, and 1 wt % of phenothiazine as a free radical scavenger. The formulation was stirred overnight in a glass 20 mL vial with a magnetic stirrer.

### Optimization of Print Conditions

Dosing parameters were first determined by printing 3 × 3 arrays comprised of 150 μm^2^ slabs with 20 μm thickness at varying laser doses, scan speeds, and voxel slicing parameters. Quality prints—evidenced by clean lines, absence of underdosed, gelatinous material, and absence of overexposed burns or bubbles—were obtained using a laser power of 15–30 mW, a scan speed of 20,000 μm/s, and a slicing distance of 0.3 μm with a 25× objective.

### Two-Photon Printing of Sinusoids

Two-photon polymerization (2PP) is a direct laser writing technique that enables high-resolution, three-dimensional microfabrication by exploiting the nonlinear absorption of femtosecond near-infrared laser pulses. It was selected over single-photon stereolithography because it offers submicron resolution and true three-dimensional free-form fabrication, allowing precise control of sinusoidal feature geometry necessary for controlled adhesion studies.^33,34^ In this work, 2PP was used to locally cure resins comprising PETMP and TTT, enabling the precise fabrication of well-defined sinusoidal metasurfaces. While the formulation includes a photobase generator and a radical scavenger, no definitive reaction mechanism is assigned; we refer to the curing process as thiol–ene photopolymerization. For each print, a droplet of prepared resins was placed on an ITO-coated, 0.7 mm thick borosilicate glass slide and loaded into a commercial 2PP printer (Nanoscribe GT) equipped with a 25× objective in the dip-in-laser-lithography (DiLL) mode. Printing was performed using a laser wavelength of 780 nm, a repetition rate of 80 MHz, and a pulse width of 100 fs. Following each print, samples were immersed in propylene glycol monomethyl ether acetate (PGMEA) to remove unreacted resin then submerged in isopropanol to remove unwanted PGMEA. After subsequent drying, samples were ready for testing.

Stereolithography (STL) files were uploaded into the NanoWrite software, and prints were made using a laser power of 20 mW and a scan speed of 12,000 μm s^–1^. Slabs were designed to be large enough to studied using compressive forces on the order of 1 N. Each print comprised of a flat slab with a square-edge dimensions ranging from 0.600 mm to 0.800 mm and a 2D sinusoidal pattern, i.e. *z* = *f*(*x*,*y*), on the top surface. Each print was designed with backing layer beneath the sinusoidal top layer, and the backing layer thickness was designed to be between 38 or 50 μm. Some samples were printed without their last printing block to aid in alignment with the top-down camera view. Images of printed metasurfaces are available as Supporting Information.

### Thermomechanical Contact Testing

All compression tests were conducted using a custom-built contact mechanics measurement system which is described in detail elsewhere.^15^ Briefly, the system comprises of a stationary glass substrate holder and a movable substrate. The movable substrate is positioned beneath, but can be pressed against, the stationary substrate. The movable substrate can be independently translated along the *x*-,*y*-, and *z*-axes using piezo stepper motors. Forces acting against the movable substrate are monitored using four, 0–5 N load cells. A thermoelectric heater is used to control the temperature of the movable substrate. The sample temperature is taken as the average of the stage temperature and the temperature of a thermocouple placed on the immovable substrate. A vertical microscope is used to monitor the plan-view as features are compressed.

Prior to compression experiments, the piezo-stage positioning system is initiated and calibrated. All surfaces, including glass slides, are cleaned with solvent to remove interfering dust particles. The stages are leveled, and the ITO glass substrate with the 2PP print is mounted onto the movable glass slide double-sided Kapton tape. For contact testing between printed surfaces and glass substrates, the print is brought into contact, and the movable stage is translated a fixed distance (typically from 0 to 30 μm) placing the sample into compression. The microscope is used to image the contact area while force sensors monitor compression forces. Next, the movable stage is retracted back to its original position to break contact and measure pull-off forces. This process is repeated three times at various contact and pull-off temperatures.

For each pull-off step, adhesion strength was determined experimentally as the maximum pull-off force measured during stage retraction after a compression, divided by the nominal contact area of the print (i.e., the edge dimension squared), and this value is referred to as the nominal adhesive strength (σ_N_). For contact testing between self-complementary pairs, the process is similar, but a second, self-complementary print is mounted on the stationary substrate with double-sided Kapton tape. The samples are aligned by rotation and translation such that the peaks of one surface register with the valleys of the other surface, and contact tests are similarly executed at various contact forces and pull-off temperatures. Sample position was collected using a commercial laser displacement sensor (Keyence, IL-030) capable of measuring to about 1 μm of stage displacement. For force–displacement measurements of **M75** surfaces against flat surfaces, sample position was calculated using the motor displacement and stress measurements based on a separate measurement of machine compliance.

### Finite Element Analysis

Surfaces were defined from STL files by transforming to a solid volume in using MATLAB software followed by creating a volumetric mesh with tetrahedral C3D4 elements using Abaqus FEM package. The glass surface used in experiments was treated as an analytical rigid surface, given the orders of magnitude difference in stiffness between the print polymer and the glass. The interaction between the two surfaces was controlled by a Surface-to-Surface contact algorithm in Abaqus that uses a hard contact interaction to define the normal displacement between the sinusoid and the glass surfaces. This minimizes the penetration of the two surfaces upon contact, enabling the normal displacement to be defined by experimental measurement. The tangential displacement behavior was controlled by a friction coefficient of 0.2 between the two surfaces, and frictional forces were determined according to the contact pressure that develops during the loading process. This value was chosen based on typical ranges reported for polymer–glass interfaces.^35^ However, the actual coefficient of friction depends on several factors including surface roughness and the state of the polymer (glassy vs rubbery). Here, our choice of 0.2 serves as an approximate value to capture some lateral resistance, while the dominant contribution to adhesion arises from normal (pull-off) forces.

### SEM Imaging

Samples were coated in 100 Å of gold and were imaged with scanning electron microscopy. SEM conditions were between 5 and 7 kV, with a 150× magnification required for **M300** metasurfaces, while 1.01 KX for highly resolved **M38**’s.

## Results and Discussion

Two-photon polymerization (2PP) printing was conducted to locally catalyze thiol-Micheal coupling between multifunctional monomers pentaerythritol tetrakis­(3-mercaptopropionate) (PETMP) and 1,3,5-triallyl-1,3,5-triazine-2,4,6­(1*H*,3*H*,5*H*)-trione (TTT). We previously showed that this photobase-initiated network consistently results in higher resolution prints with lower print error compared to radical-initiated formulations.^31^ In the current study we used a commercially available photobase initiator, 9-anthrylmethyl *N*,*N*-diethylcarbamate, which is photoactive at 395 nm and has a high quantum efficiency.^36^ This particular resin formation shows excellent print properties when printed using a laser power of 20–30 mW with a 25× objective. While this dose may be too high for commonly used resins, it aligns well with the higher power threshold needed for the stoichiometrically balanced mixture of step-growth monomers.

Four different designs of flat slabs with sinusoidal surfaces on their top side were printed. Stereolithography (STL) designs were generated by the function

1 f(x,y)=Asin(2πωx)cos(2πωy)+b

where *A* is the amplitude of the sinusoidal surfaces, ω is the spatial frequency obtained by dividing number of sinusoids *n* to the base edge dimension *w*, and *b* is an offset to allow for a supporting base layer.

Figure 1 shows a schematic of the printing process, chemical structures of resin components and metasurface designs and prints. The sample name indicates the metasurface period, in microns, of the sinusoidal surface along a lateral dimension. Design dimensions and measured print dimensions are summarized in Table 1. While the data indicate that printed dimensions agree with design dimensions quite well, we do not have a direct measurement of surface area. Mathematically, the top surface area *S* of each print can be calculated using the sinusoidal function *f* in eq 1 by

2 $$S=\iint \sqrt{{\left[\frac{\partial f}{\partial x}\right]}^{2}+{\left[\frac{\partial f}{\partial y}\right]}^{2}}dA$$

which exceeds the projected (areal) surface area by an enhancement factor *M*, also shown in Table 1. To quantify the expected increase in surface area, the enhancement factor, *F*, is calculated as the ratio of the theoretical sinusoidal surface area to the projected area. For example, the sinusoidal surface **M75** enhances the top surface area by a factor of about two.

**Figure 1:**
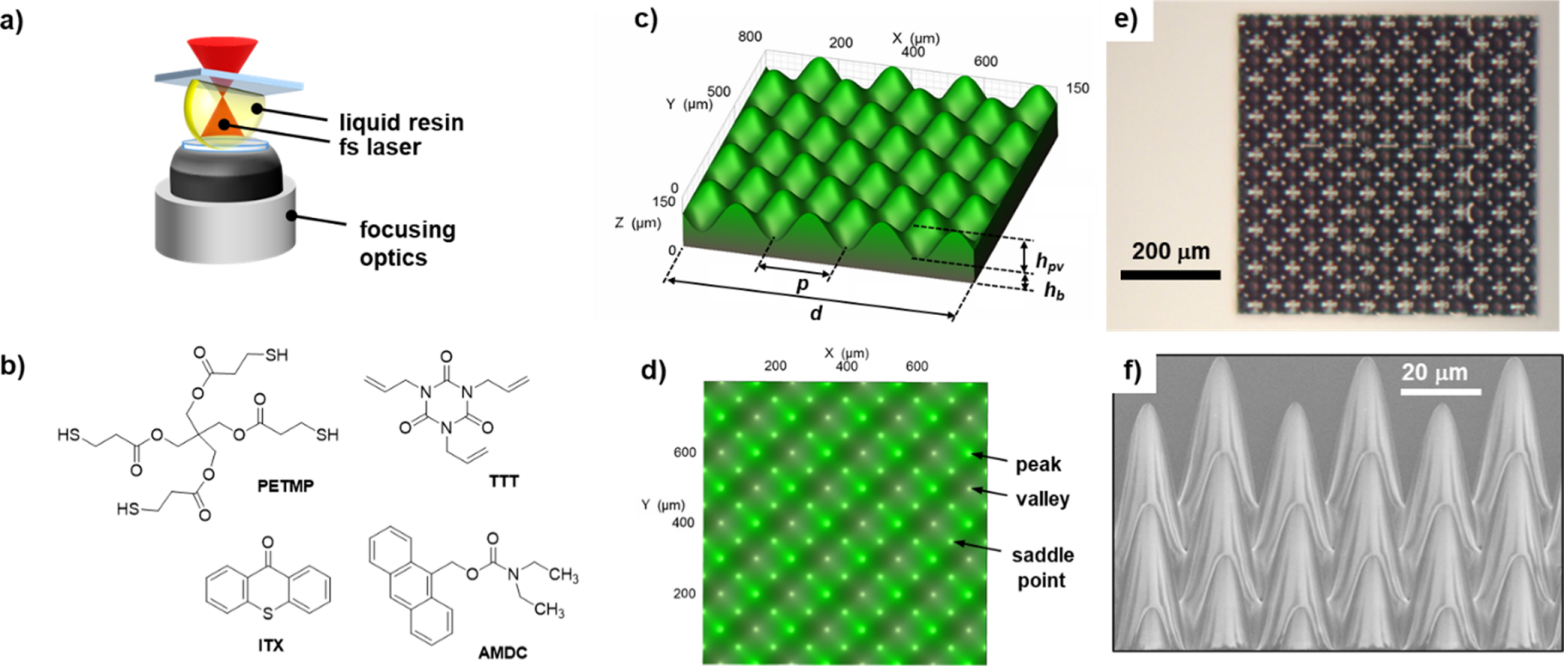
Design and printing of sinusoidal surfaces: (a) schematic showing two-photon printing within a liquid resin droplet; (b) chemical structures of resin components, (c,d) high resolution images of designed **M200** structure from tilted and top-down projections, (e) optical microscope image of two-photon printed **M75** surface, and (f) SEM image of **M38** surface.

**Table 1:** Dimensions of Square Slabs With Sinusoidal Metasurfaces Surfaces Printed Using Two-Photon Polymerization

design/print name	base edge dimension (μm)	base layer thickness, *h*_b_ (μm)	peak-to-valley height, *h*_PV_ (μm)	designed period, *p* (μm)	measured period, *p*_m_ (μm)^a^	surface area enhancement factor, *F* ^b^
**M300**	600	37.5	76.6̅	300	290	1.09
**M200**	800	50	100	200	192	1.33
**M75**	600	50	100	75	72.2	2.00
**M38**	600	50	100	37.5	37.4	3.57

a SEM images of surfaces are available as Supporting Information (Figure S1).

b The enhancement factor is calculated as the ratio of *S* from eq 2 to the projected surface area.

To investigate thermomechanical behavior, metasurface **M75** was studied using temperature-controlled compression against a rigid, glass surface, and results are shown in Figure 2. The **M75** print was subjected to increasing levels of compressive displacement (0–30 μm of motor displacement) at an uncorrected strain rate of 1 μm/s, as shown in Figure 2a–c for experiments conducted at different temperatures. This relatively slow strain rate was chosen to approximate quasi-static conditions and to minimize inertial and thermal effects. While additional rate-dependent studies would offer deeper understanding of viscoelastic effects and potential thermal softening, they are beyond the scope of the current work.

**Figure 2:**
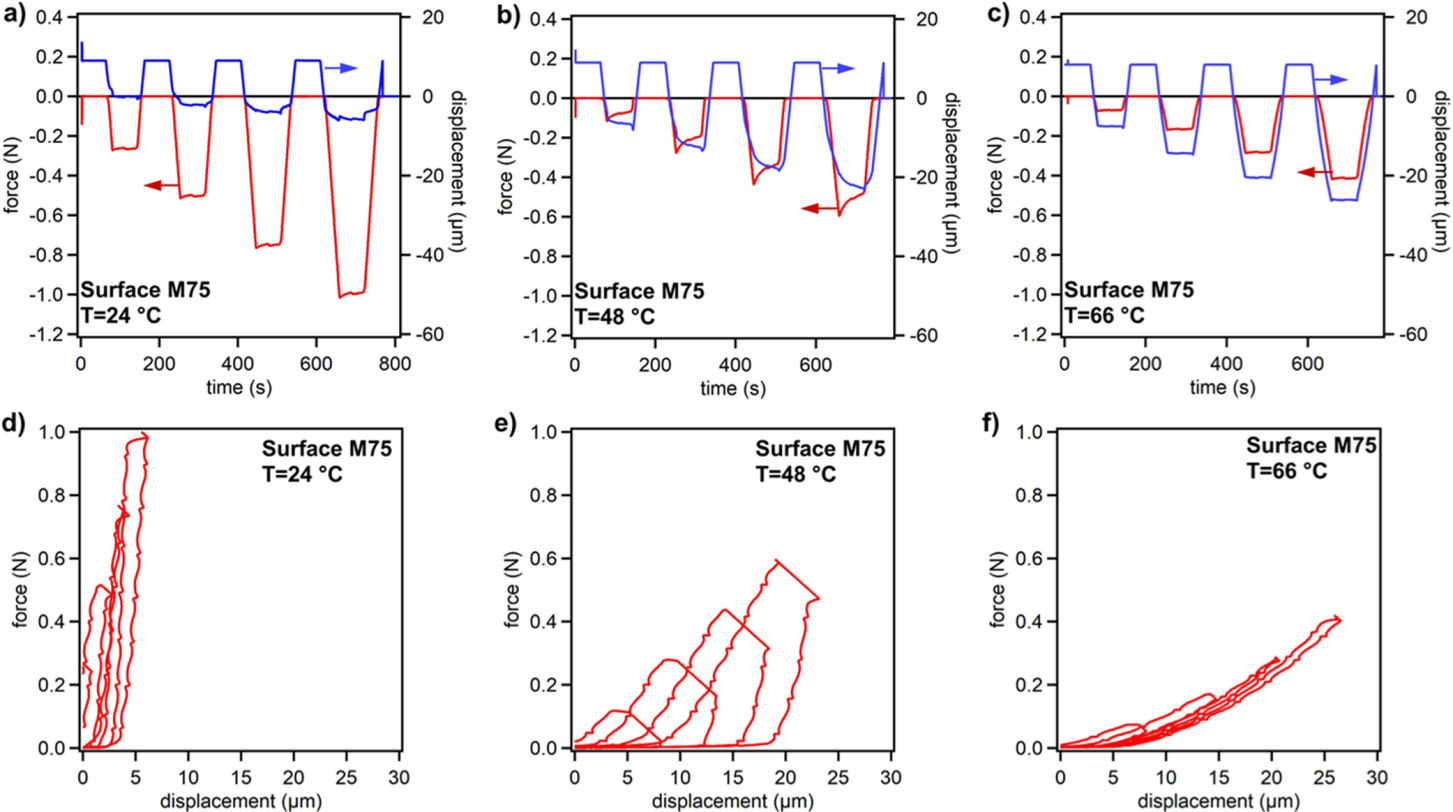
Force–displacement data for metasurface **M75** pressed against a glass substrate taken at different temperatures: (a,d) 24 °C, (b,e) 48 °C, and (c,f) 66 °C. In panels (a–c) measured forces and displacements are plotted against time, with negative forces indicating compression. In panels (d–f) measured compressive forces are plotted as positive values against displacement.

The temperature-dependent mechanical behavior of the printed shape-memory polymer is clearly reflected in the force–displacement responses measured at different temperatures. Results confirm that at room temperature (24 °C), the sample behaves as a stiff, glassy material with minimal deformation and negligible hysteresis, consistent with its high modulus. At 48 °C, near the glass transition temperature (*T*_g_), significant hysteresis is observed in the force–displacement curves, indicating viscoelastic relaxation during compression and unloading. This behavior suggests that the material begins to transition, allowing partial rearrangement of the polymer network. At 66 °C, well above *T*_g_, the sample exhibits a soft, rubbery response with lower force levels and nearly reversible deformation, reflecting rapid stress relaxation and reduced resistance to compression. The progressive decrease in force and increase in compliance with rising temperature clearly demonstrate the switchable mechanical properties of the shape-memory polymer, which are critical for controlling adhesion in subsequent experiments. These trends are illustrated in Figure 2a–f, showing the corresponding force–displacement behavior and relaxation dynamics at each temperature.

Finite element modeling (FEM) was used to fit the force–displacement data presented in Figure 2 and to relate deformation behavior to the material properties of printed metasurfaces. We assumed that the material properties are independent of topology and selected metasurface **M75** as the model geometry. FEM results are summarized in Figure 3. As shown in Figure 3a, tetrahedral elements were used to mesh the geometry, and the model was pressed against a rigid surface to replicate the experimental setup of metasurface **M75** contacting a glass substrate. A contact coefficient of friction of 0.2 was assumed. The prescribed displacement in the *z*-direction was derived directly from the experimental position–time data in Figure 2a,c, corresponding to room temperature and 66 °C, to capture behavior in the glassy (hard) and rubbery (soft) states.

**Figure 3:**
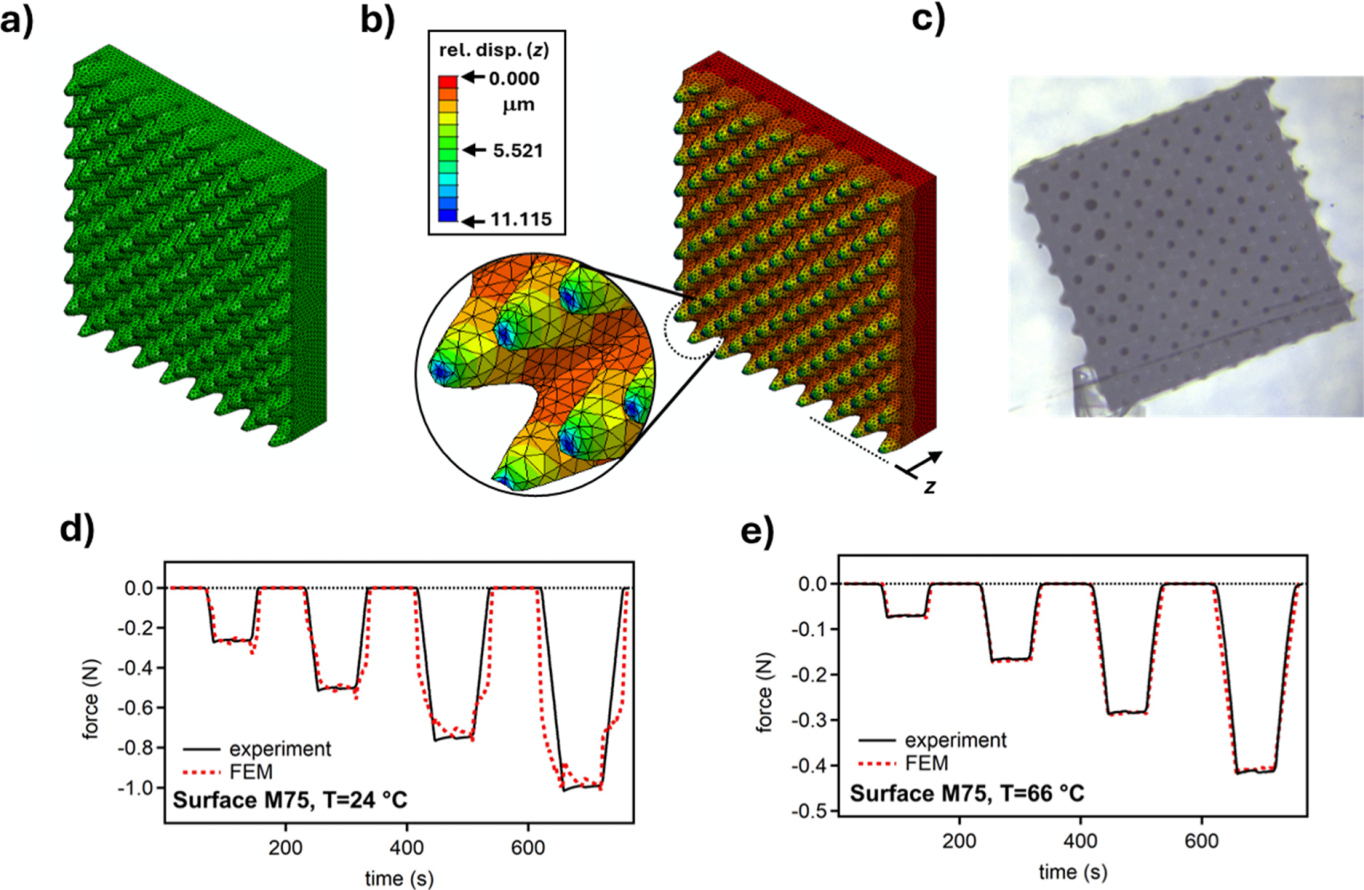
FEM modeling of metasurface **M75** to examine deformation response and elastic modulus: (a) image shows FEM model using linear tetrahedral elements defining the metasurface; (b) image showing relative surface deformation in the normal direction (*z*) that closely match (c) the experimental observation of a top-down view of a compressed print with an edge dimension of 600 μm, serving as a reference for scale; and (d,e) plots of force versus time data obtained from FEM in comparison to the experimental data at room temperature and at 66 °C. Negative force indicates compressive force.

Figure 3b shows a contour plot of deformation in the normal (*z*) direction, illustrating how the sinusoidal features deform during contact with the glass substrate. The contour plot closely agrees with the experimentally observed top-down image shown in Figure 3c: both exhibit flattening of the sinusoidal peaks, lateral spreading of the contact regions, and similar edge deformation patterns. This qualitative match between simulated and experimental deformed shapes supports the validity of the FEM model in capturing real contact mechanics.

We varied Young’s modulus in the model to match the simulated force-time data to the experimental force curves in Figure 2a,c. The polymer was assumed to be linearly elastic and incompressible. Modeling results indicate that, at room temperature, the material behaves as a linear elastic solid with negligible relaxation, as shown in Figure 3d. The larger variation observed in the modeling data at room temperature is attributed to fluctuations in the displacement input from the experiment (Figure 2a). Nevertheless, the FEM approach successfully matches the steady-state force levels at room temperature. The same procedure was used to analyze behavior at 66 °C, incorporating a viscoelastic model defined by a Prony series to account for stress relaxation. Figure 3e shows that the high-temperature simulation results align very well with experimental data and exhibit much less force variation, owing to smoother displacement inputs (Figure 2c). Based on our FEM modeling, we estimated an apparent elastic modulus of 253 MPa at room temperature and 23 MPa at 66 °C. These values are intended to capture the effective compressive response of the printed metasurfaces in contact experiments and are not meant to represent bulk or tensile properties.

To study adhesion, printed surfaces were compressed against rigid, glass surfaces and against self-complementary surfaces following the protocol outlined in Figure 4. Cold and hot compressions involve pressing and decompressing sinusoidal features by controlled stage translation while measuring the resulting force at different temperatures. For shape-memory compressions, features are first compressed at a temperature above the sample’s glass transition temperature (*T* > *T*_g_); cooled beneath 30 °C while under compressive forces; and, when sufficiently cooled, the substrate is retracted. Postshape-memory (PSM) compressions involve first programming the features by compressing them while *T* > *T*_g_, then cooling below *T*_g_ while maintaining compression, followed by stage retraction at *T* < *T*_g_. The programmed features are subsequently subjected to cold–compression cycles during which stress is measured.

**Figure 4:**
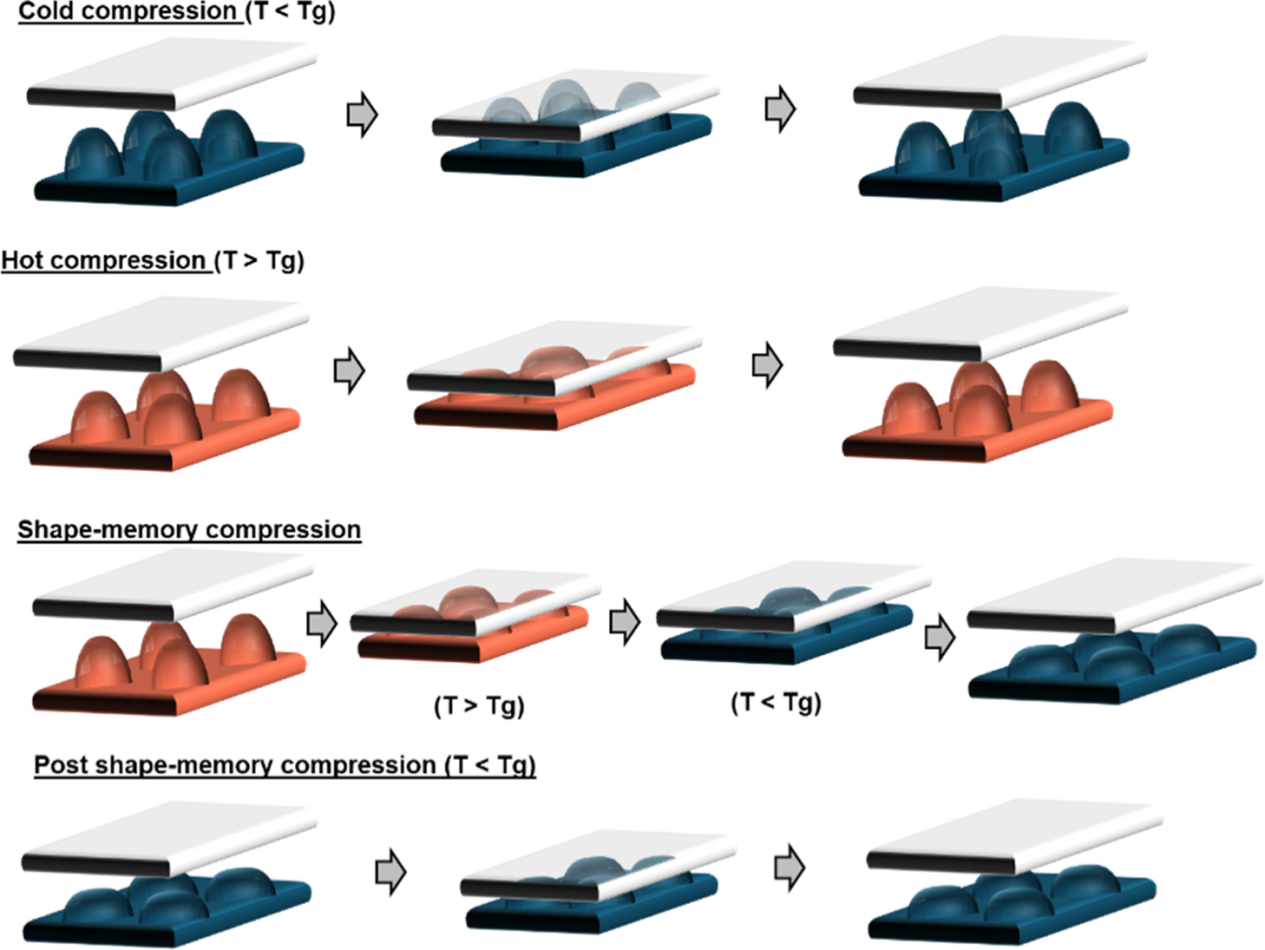
Experimental protocols to study adhesion between printed meta-surfaces and an opposing flat surface.

### Adhesion Between Flat Surfaces

Figure 5 shows an example of force–displacement data and image analysis during cold compression and pull-off of a printed 1 mm × 1 mm flat slab compressed against a rigid, glass surface. Immediately prior to detachment, the print experiences a maximum of about 0.2 N of tension corresponding to a nominal “pull-off” or adhesive strength σ_*n*_ of 0.2 MPa based on the projected print area of 1.0 mm^2^. Further, variation of the actual contact area with time *A*_a_(*t*) could be determined by analysis of video images obtained at a high frame rate (see Video S1). The tension acting on the contact area immediately before detachment corresponds to an actual pull-off stress or adhesive strength of σ_a_ = 0.42 MPa. The total pull-off work expended to detach the two surfaces could be calculated directly from acquired data as

$$W_{tot}=\int F\left(t\right)\left(\frac{dx\left(t\right)}{dt}\right)dt$$

where *x*(*t*) and *F*(*t*) are measured displacements and force. This work, calculated numerically from the data shown in Figure 5c, corresponds to a value of 3 × 10^–4^ mJ/mm^2^.

**Figure 5:**
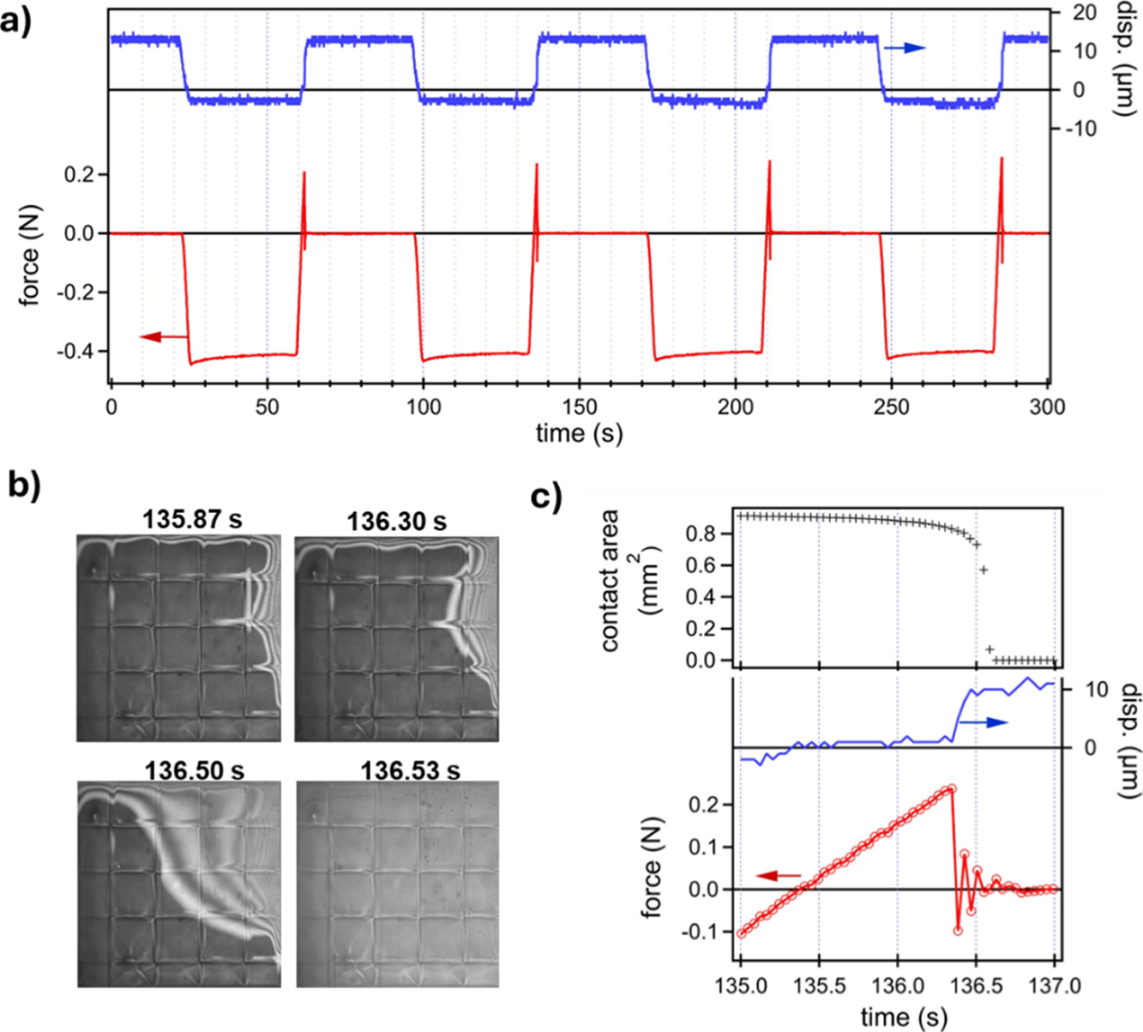
Experimental data for room temperature compression and detachment of a printed flat slab (1 mm × 1 mm) against a glass interface: (a) measured displacement *x*(*t*) and force *F*(*t*) for four cycles; (b) sample video images acquired at 30 frames per second showing contact area as darker pixels; and (c) time-resolved plots of contact area *A*(*x*), displacement *x*(*t*), and force *F*(*t*) as the print-glass interface breaks. The square grid appearing in video images is stitching of overlapped regions in direct laser written print. Negative force indicates compressive force.

This total pull-off work can be broken into two parts

$$W_{tot}=W_{str}+W_{\mathrm{int}}$$

where *W*_str_ is the work expended to elastically stretch the printed slab, and *W*_int_ is the interfacial work to break short-range, adhesive interactions. Provided values for Young’s modulus and interfacial energies, *W*_str_ and *W*_int_ can be directly calculated from experimental data *x*(*t*), *F*(*t*), and *A*(*t*) by

$$W_{str}=\int E\frac{x}{d}A_{a}\left(t\right)\left(\frac{dx\left(t\right)}{dt}\right)dt$$

and

$$W_{\mathrm{int}}=\int \left(\gamma_{p}+\gamma_{g}-\gamma_{pg}\right)\left(\frac{dA_{a}\left(t\right)}{dt}\right)dt$$

where γ_p_ and γ_g_ are the interfacial energies of free print and glass surface, and γ_pg_ is the interfacial energy of the contacted surface. Using our estimate of Young’s modulus from finite element analysis (E ∼ 253 MPa) and typical values of interfacial surface energies (γ_p_ ≈ γ_p_ ≈ γ_pg_ ≈ 50 mJ/m^2^) with experimental data (*x*(*t*), *F*(*t*), and *A*(*t*)) one finds that the total pull-off work is dominated by stretching energy. For example, for the print-glass experiment shown in Figure 5, the calculated stretching work per initial contact area is 3 × 10^–3^ mJ/mm^2^, and the calculated interfacial work in on the order of 10^–5^ mJ/mm^2^. This measurable interfacial work is small and is typically within the experimental error of the measured total work; and, therefore, for the remainder of the manuscript we will report measured interfacial adhesive strength σ as determined by the maximum observed pull-off force to evaluate adhesion of printed metasurfaces. Furthermore, since the true area of contact cannot clearly be measured for contacting metasurfaces, values of the nominal pull-off strength σ_*n*_, based only on the nominal contact area, are reported.

### Adhesion Involving Printed Metasurfaces Opposing Flat, Glass Surfaces

Printed metasurfaces were pressed against flat, glass surfaces, and the resulting adhesion strength was measured upon stage retraction. Experiments were performed at room temperature, elevated temperature (63 °C), and following shape-memory programming, as outlined in Figure 4. All metasurfaces showed negligible adhesive pull-off forces when pressed against flat glass surfaces at room temperature and at 63 °C (see Supporting Information, Figure S2). These compressions require significant elastic deformation of the metasurface, as modeled by finite element analysis in the previous section, and the imposed deformation aids in detachment at all temperatures.

Metasurface **M38**, consisting of a 600 μm × 600 μm slab with a sinusoidal surface, exhibits a nominal adhesive pull-off force of 0.2 N when tested against flat glass using the shape-memory protocol, and an example experimental data set is shown in Figure 6. However, when the sample is subsequently heated to 60 °C, the adhesion is completely switched off. This result demonstrates how high frequency 3D printed metasurfaces such as **F16** can be thermo-mechanically programmed to temporarily enhance adhesion.

**Figure 6:**
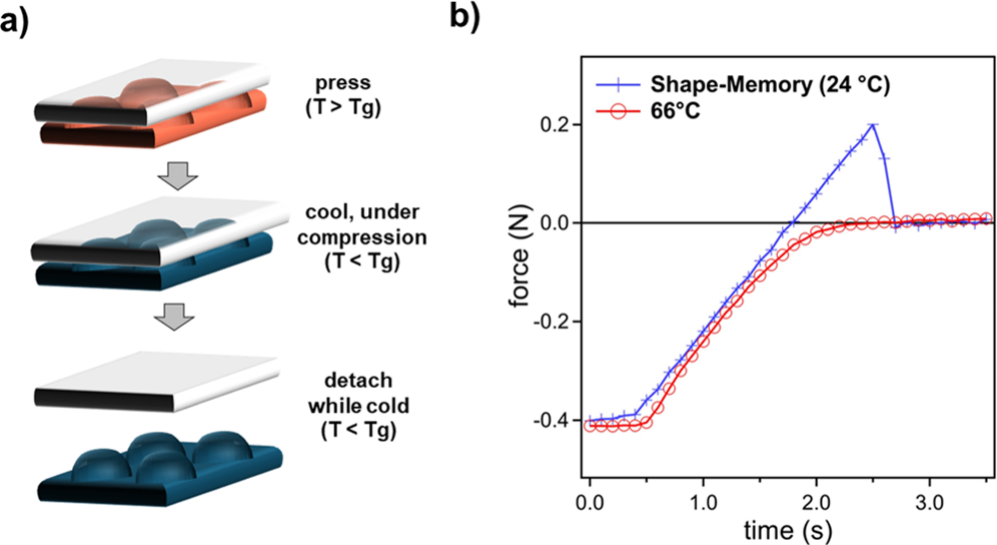
Shape-memory assisted adhesion of metasurface **M38** against a flat substrate: (a) shape-memory programming of the metasurface involved cooling while under a compressive load of 0.4 to 2.0 N before detachment; and (b) experimental force–displacement data during detachment following shape-memory programming (blue, “+”) and after heating to 60 °C (red, “o”). The inset to (b) shows a top-down view of the metasurface before detachment, and the sample’s edge dimension is 600 μm. Negative force indicates compressive force.

The observed shape-memory enhancement of adhesion is primarily attributed to shape stabilization of flattened features.^15^ When metasurfaces are compressed above the glass transition temperature (*T* > *T*_g_), individual sinusoidal features deform into flatter shapes that conform more closely to the opposing surface, effectively increasing the real contact area and associated adhesive forces. Upon cooling below Tg while under compression, these deformed shapes are fixed as they transition into a glassy state, maintaining elevated contact area and interfacial adhesion forces upon retraction. However, reheating the surfaces above Tg allows the features to release stored elastic energy, which aids in breaking interfacial adhesive forces, thereby lowering adhesive pull-off strength. This mechanism provides a straightforward thermal trigger for controlling adhesion, which is particularly valuable for applications requiring on-demand assembly and disassembly of microscale components.

In contrast to metasurface **M38**, metasurfaces **M300**, **M200**, and **M75** showed negligible adhesive strength, even when processed using the shape-memory protocol, and the adhesive properties of metasurface **M38** are attributed to the relative ease of deforming densely packed, small features. A scaling argument based on Johnson–Kendall–Roberts (JKR) theory suggests that soft surfaces with many small features should exhibit higher pull-off forces than surfaces with fewer, large features. The JKR model predicts that the pull-off force for a single elastic sphere of radius *R* in contact with a flat, rigid substrate scales linearly with sphere radius, *F*_PO_ ∼ *R*. The sinusoidal metasurfaces have protruding features that can be approximated as spheres with radii that scale with surface frequency as *R* ∼ ω^–1^. Since the number density of features scales as *N* ∼ ω^2^, the total pull-off force of features acting in parallel should scale linearly with ω. This simple argument explains why metasurface **M38** exhibits measurable adhesion and metasurfaces with lower surface frequencies do not.

### Adhesion Involving Opposing, Self-Complementary Metasurfaces

For experiments involving two, opposing printed self-complementary surfaces, the measured pull-off adhesion greatly depends on spatial frequency. For **M300** and **M200** metasurfaces, little to no adhesion was observed (see Supporting Information, Figures S3 and S4), and this is attributed to a combination of (i) surface roughness and (ii) interference or mismatch of the periodic print bed/stitching features with the periodic metasurface. These factors lead to higher levels of elastic deformation as contact is made. Further optimization of printing parameters (e.g., laser power, scan speed, slicing distance) or alternative resins could enhance surface fidelity and reduce roughness thereby improving conformal contact for low frequency surfaces.

In contrast to low frequency surfaces, significant pull-off forces were measured following compression of self-complementary **M75** and **M38** metasurfaces as displayed in Figure 7, and the corresponding raw force-versus-time data and videos are provided as Supporting Information (Figures S5–S8). Self-complementary compressions of **M75** metasurfaces reveal measurable pull-off adhesion at room-temperature. Figure 7a compares the nominal adhesive strength of two opposing **M75** metasurfaces to that of two opposing flat prints at different loading pressures. At room temperature, compression of the **M75** metasurfaces results in only partial contact, as seen in Figure 7b, which accounts for the lower observed pull-off forces relative to flat surfaces. However, shape-memory programming of the **M75** metasurfaces promotes improved contact by deforming features to better conform and interlock during compression above Tg, followed by cooling, leading to higher adhesive strength. Figure 7c shows excellent contact between two previously programmed **M75** surfaces during a postshape-memory adhesion experiment, and nominal pull-off strengths are nearly double those of flat surfaces.

**Figure 7:**
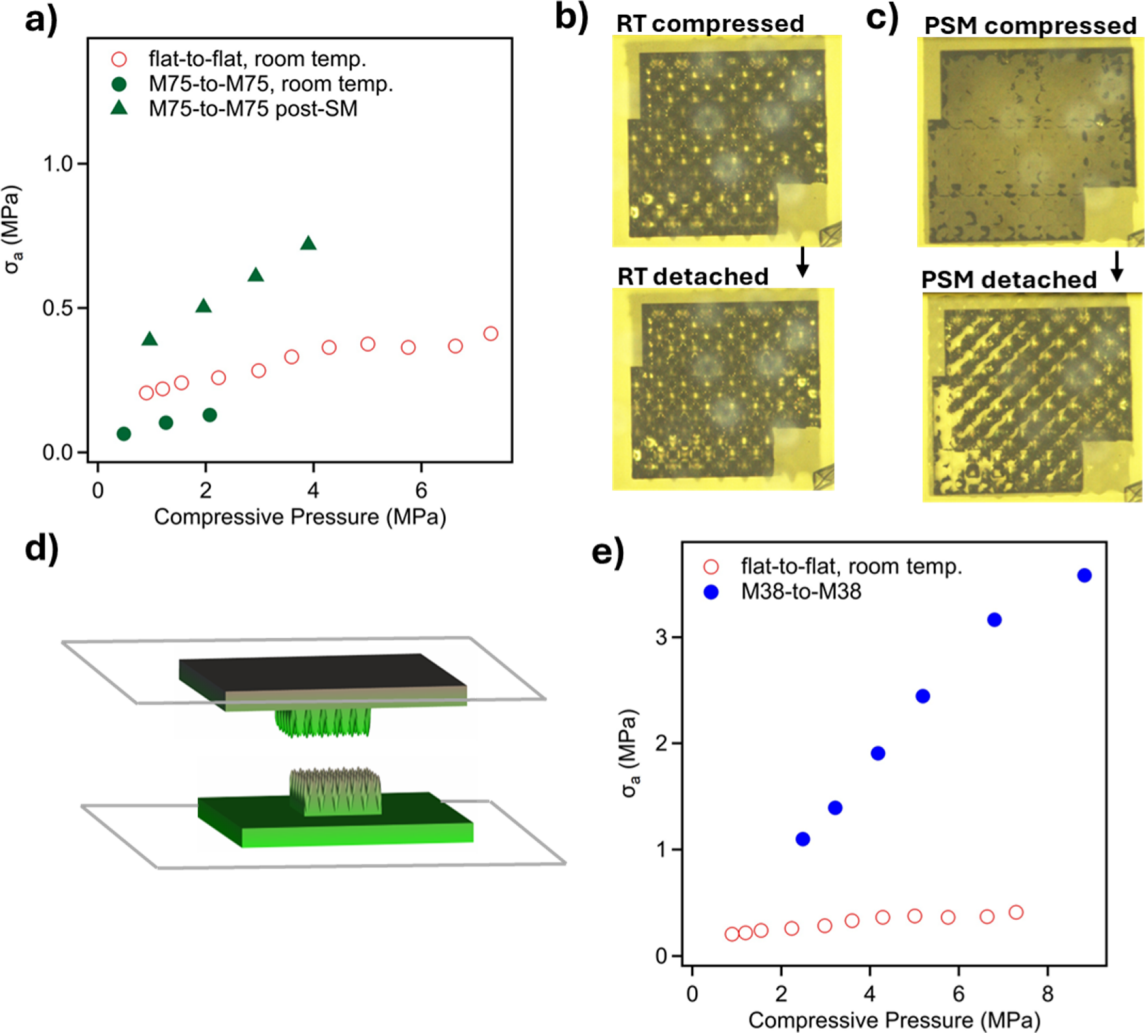
Adhesion self-complementary metasurfaces: (a) comparison of nominal pull-off adhesion strength between two **M75** metasurfaces and two flat surfaces; (b,c) top-down images of two opposing **M75** metasurfaces before and after delamination showing contact at room temperature and following shape-memory programming; (d) image of modified **M38** metasurface with reduced contact area; and (e) comparison of nominal pull-off adhesion strength between two **M38** metasurfaces and two flat surfaces at room temperature. The image scale in (b,c) is defined by the largest print’s (gray area) edge dimension of 600 μm.

Although no significant changes in adhesion strength or visible surface damage were observed over the tested cycles (up to ∼12), potential long-term fatigue or gradual wear of surface features could impact performance in high-cycle applications. This consideration is important for practical use and motivates further evaluation of durability under extended cycling.

Self-complementary adhesion experiments on 600 μm × 600 μm prints of metasurface **M38** demonstrated exceptionally strong adhesion, such that, after multiple attempts, failure consistently occurred at the weaker print-glass interface rather than between the metasurfaces themselves. Therefore, modified monoliths comprising of an underlying 600 μm × 600 μm flat region connected to a 200 μm × 200 μm textured regions were printed as shown Figure 7d. The textured regions each had the same structural factors (wavelength, amplitude) as the original **M38** surface, but with fewer features. The nominal pull-off adhesive strength between these two surfaces is plotted as a function of applied load in Figure 7e, and the adhesive strength exceeds the strength of opposing flat surfaces by nearly an order of magnitude. A video showing detachment of two prints adhered at the fractured region is available as Supporting Information (Video S4).

Compared to the other metasurfaces with lower surface frequencies, complementary compression of two **M38** metasurfaces demonstrated the greatest amount of adhesive pull-off force. This large adhesive strength is partly attributed to the **M38** metasurface having about three times the surface area of the flat sample, allowing for greater interfacial forces. Additionally, jamming may play a role because printed features may deviate slightly from their designed shape due to print inaccuracies. If a sinusoidal feature is wider than its opposing valley, then the feature may be laterally compressed, leading to additional traction forces during pull-off. Interlocking features that require elastic deformation to form a mated surface will be the subject of a future investigation.

## Conclusions

Two-photon polymerization uniquely allows for highly customizable features at length scales closer to the natural, nanometer length scale of intermolecular van der Waals interactions. We demonstrated that direct laser writing is an effective tool to create high-surface area, self-complementary metasurfaces for improved adhesion. Printed flat slabs and sinusoidal metasurfaces show repeatable adhesion over several compression cycles. Further, the use of resins that can be cured into shape-memory networks is valuable because small scale surface roughness can be temporarily removed, further enhancing adhesive strength.

Sinusoidal metasurfaces show little adhesion to flat glass substrates owing to their elastic deformation, however stronger adhesion was observed between self-complementary metasurfaces, particularly at high surface frequencies. Moderate adhesive strength (0.1 to 1 MPa), comparable to that of flat surfaces, was observed following compression of self-complementary **M75** metasurfaces but was limited by incomplete contact between features. In contrast, an adhesive strength exceeding 3 MPa was measured following compression of two self-complementary **M38** metasurfaces with a spatial frequency of 26.6̅ mm^–1^; this higher strength is attributed to more complete interlocking and increased real contact area, highlighting the critical role of surface frequency in optimizing self-complementary adhesion.

Printed metasurfaces with shape-memory have the potential to greatly simplify manufacturing of complex, heterogeneous devices by facilitating alignment and assembly of microscale parts. Furthermore, assembled surfaces can be triggered for facile disassembly using a shape-memory stimulus. The ability to reduce adhesion by heating above Tg allows components to be separated easily without mechanical prying or solvents, minimizing damage and enabling repeated reuse. After detachment, surfaces can return to their original shape and be reprogrammed for subsequent adhesion cycles, highlighting their potential in recyclable or reconfigurable systems. Such triggered disassembly could be especially useful for recovering and reusing system components or valuable materials such as precious metals, paving the way for improved recycling.

## Supplementary Material



## Notes

Images of two-photon printed metasurfaces, additional force and force–displacement data from adhesion experiments, and description of uploaded videos (PDF)
