# Supplementary material for: Adhesion of Self-Complementary, Sinusoidal Surfaces Fabricated Using Two-Photon Polymerization
Source: ACS Appl Polym Mater. 2025 Sep 25;7(19):13276–85. doi: 10.1021/acsapm.5c02773 (PMC12519446; doi:10.1021/acsapm.5c02773)
Supplement: Supplementary file 1 [file ap5c02773_si_001.pdf]

Supporting Information

Adhesion of Self-Complementary, Sinusoidal Surfaces Fabricated using Two-Photon Polymerization

Madelyn P. Jeske[1,2], Hannan Wang[3], Hesam Askari[3], David R. Harding[1,2], Mitchell Anthamatten[1,2] *

1. Department of Chemical Engineering, 4306 Wegmans Hall, University of Rochester, Rochester, NY 14627

2. Laboratory for Laser Energetics, 250 East River Road, University of Rochester, NY 14623

3. Department of Mechanical Engineering, 235 Hopeman Bldg., University of Rochester, Rochester, NY 14627

* e-mail: mitchell.anthamatten@rochester.edu

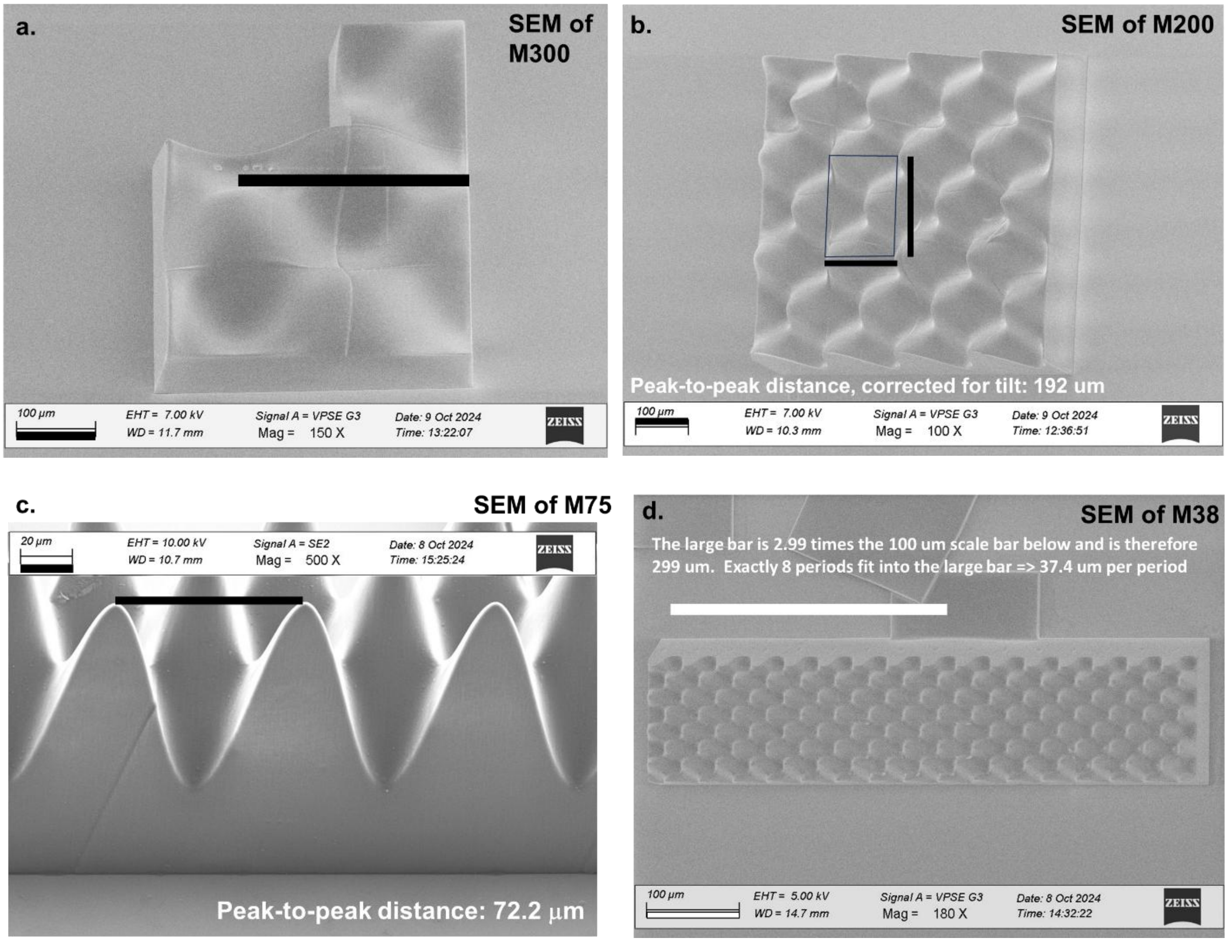

Figure S1. SEM images of two-photon printed metasurfaces: (a) M300; (b) M200; (c) M75; (d) M38.

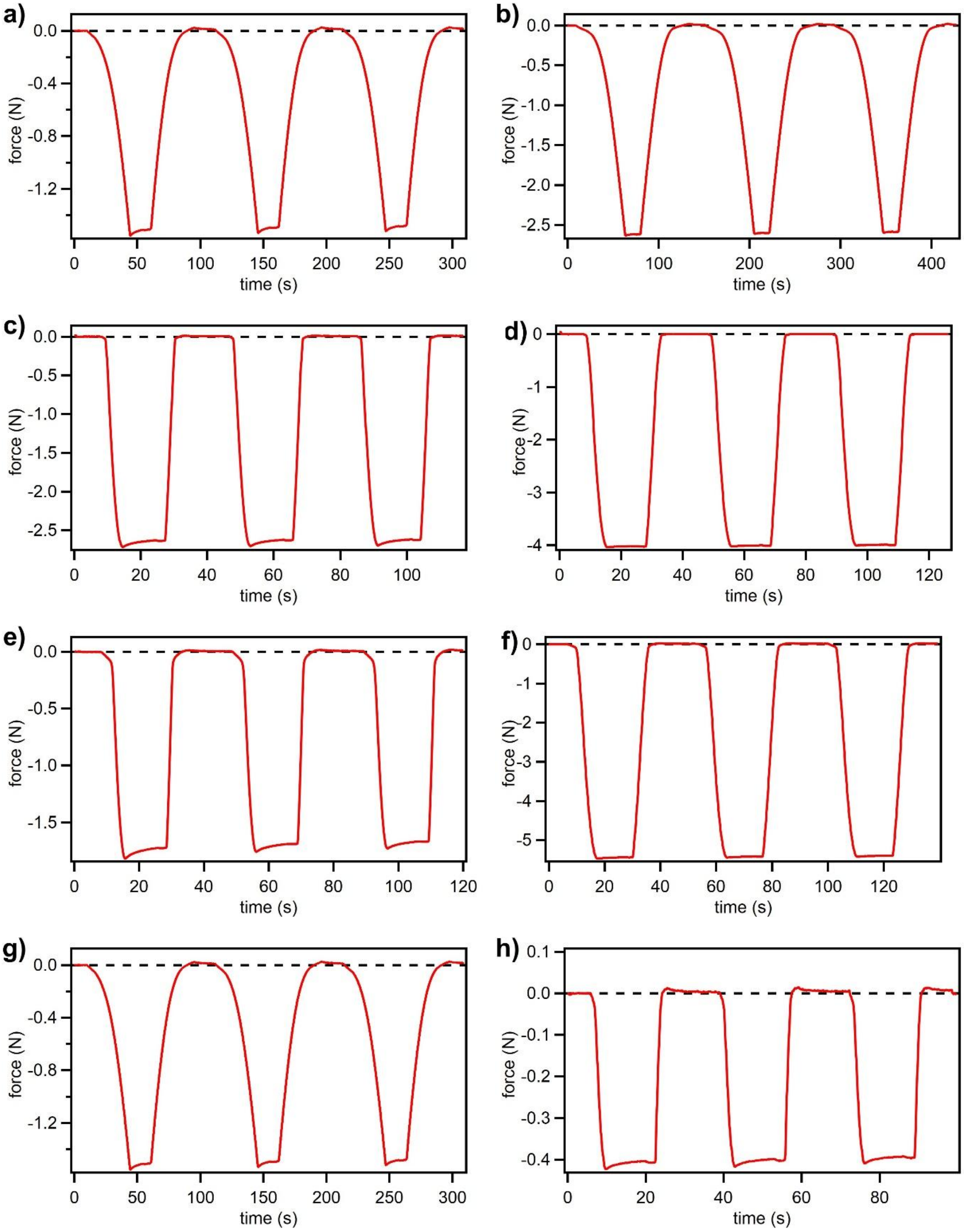

Figure S2. Measured force versus time for different metasurfaces pressed against a glass slide: metasurface **M300** at (a) room temperature and (b) at 66 °C; metasurface **M200** at (c) room temperature and (d) at 66 °C; metasurface **M75** at (e) room temperature and (f) at 66 °C; metasurface **M200** at (g) room temperature and (h) at 66 °C.

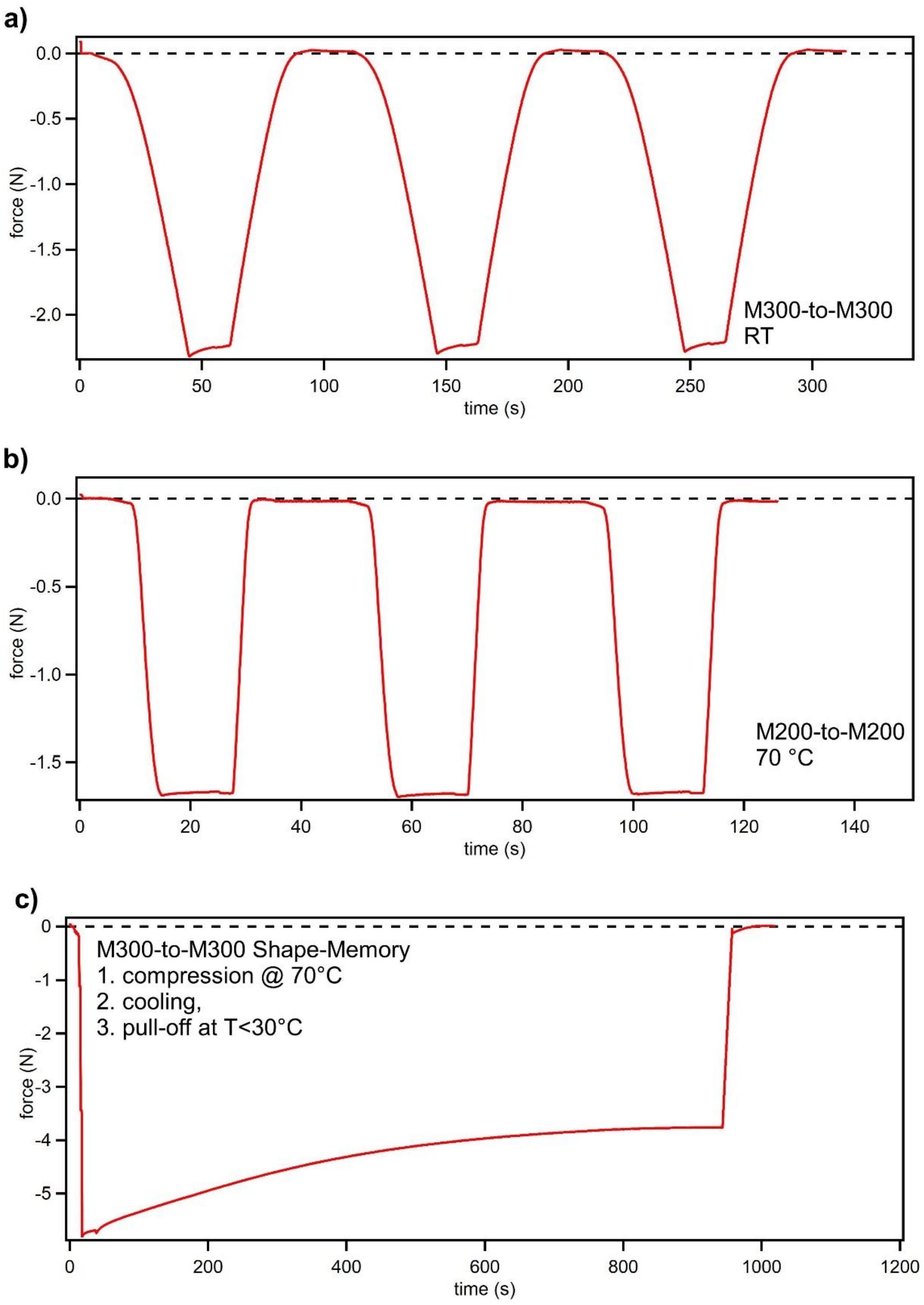

Figure S3. Measured force versus time for two opposing **M300** metasurfaces: (a) room temperature and (b) at 70 °C; and (c) following the shape-memory protocol**.**

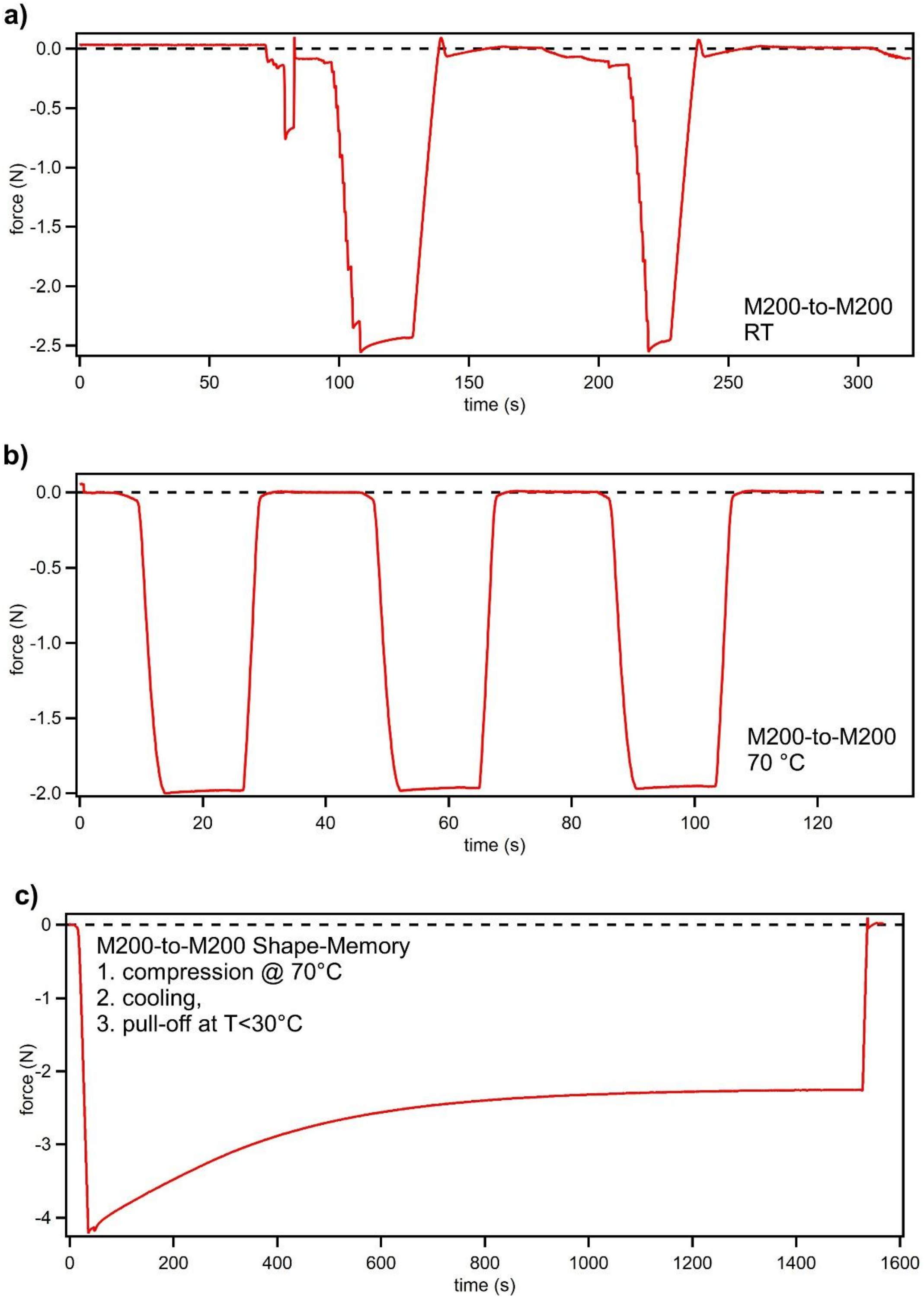

Figure S4. Measured force versus time for two opposing **M200** metasurfaces: (a) room temperature and (b) at 70 °C; and (c) following the shape-memory protocol**.**

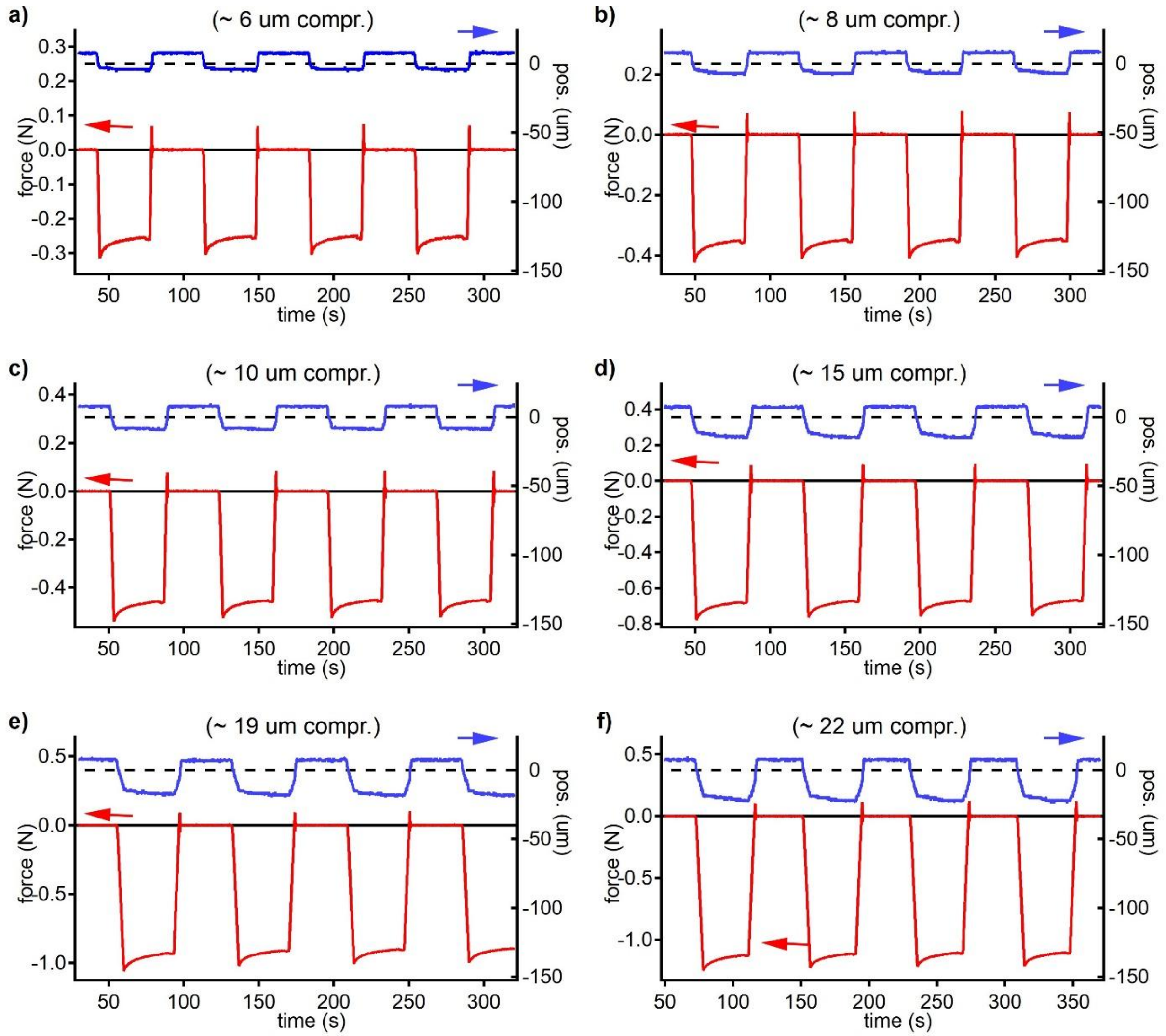

(continued on next page)

Figure S5. Measured force and distance versus time for two opposing **flat** prints at various compressions: (a) 6 μm; (b) 8 μm; (c) 10 μm; (d) 15 μm; (e) 19 μm; (f) 22 μm; (g) 25 μm; (h) 29 μm; (i) 33 μm; (j) 35 μm; (k,l) 37 μm. The adhesive strengths from these measurements reported in Figure 7.

(continued from previous page)

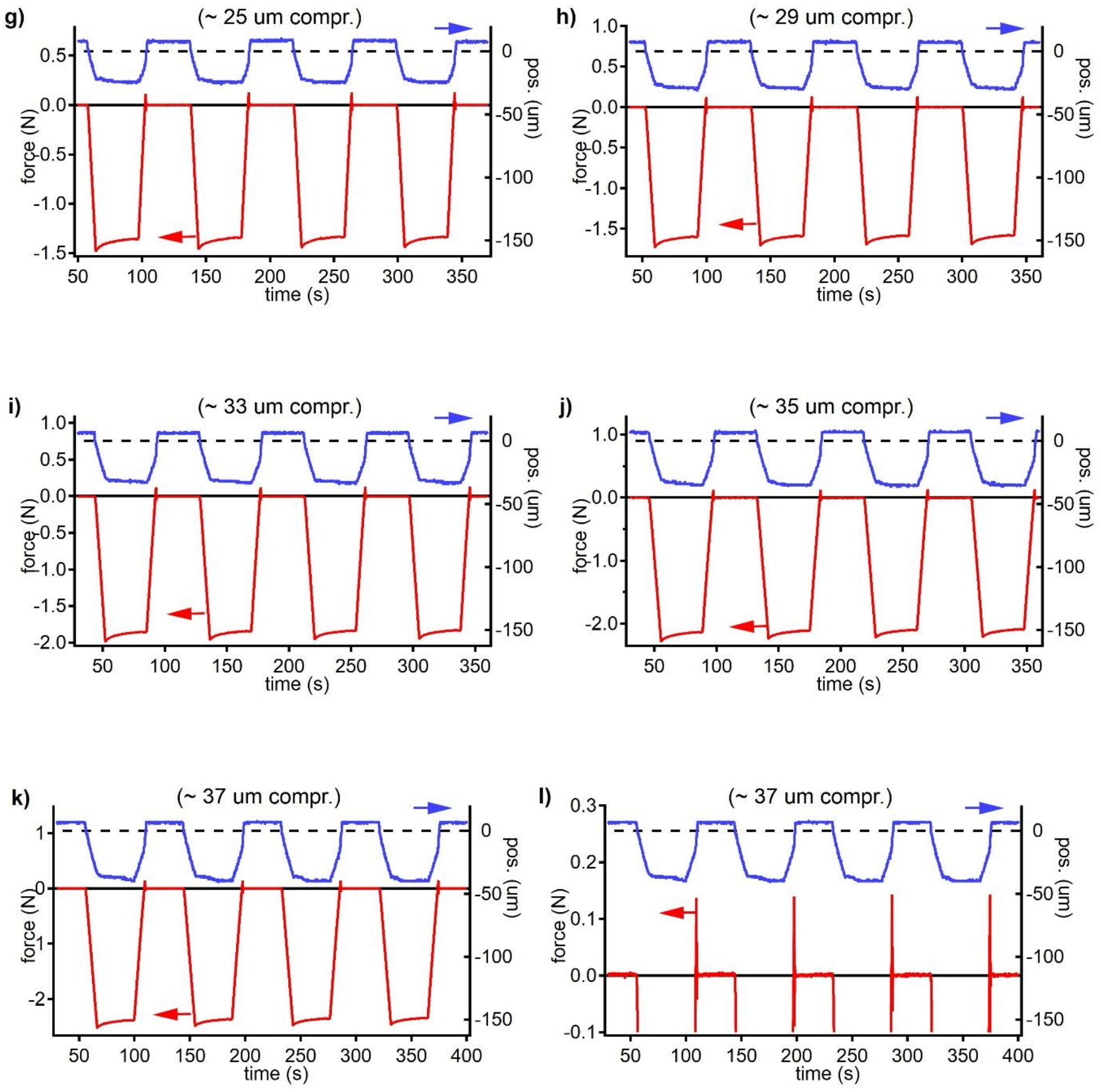

Figure S5. Measured force and distance versus time for two opposing **flat** prints at various compressions: (a) 6 μm; (b) 8 μm; (c) 10 μm; (d) 15 μm; (e) 19 μm; (f) 22 μm; (g) 25 μm; (h) 29 μm; (i) 33 μm; (j) 35 μm; (k,l) 37 μm. The adhesive strengths from these measurements reported in Figure 7.

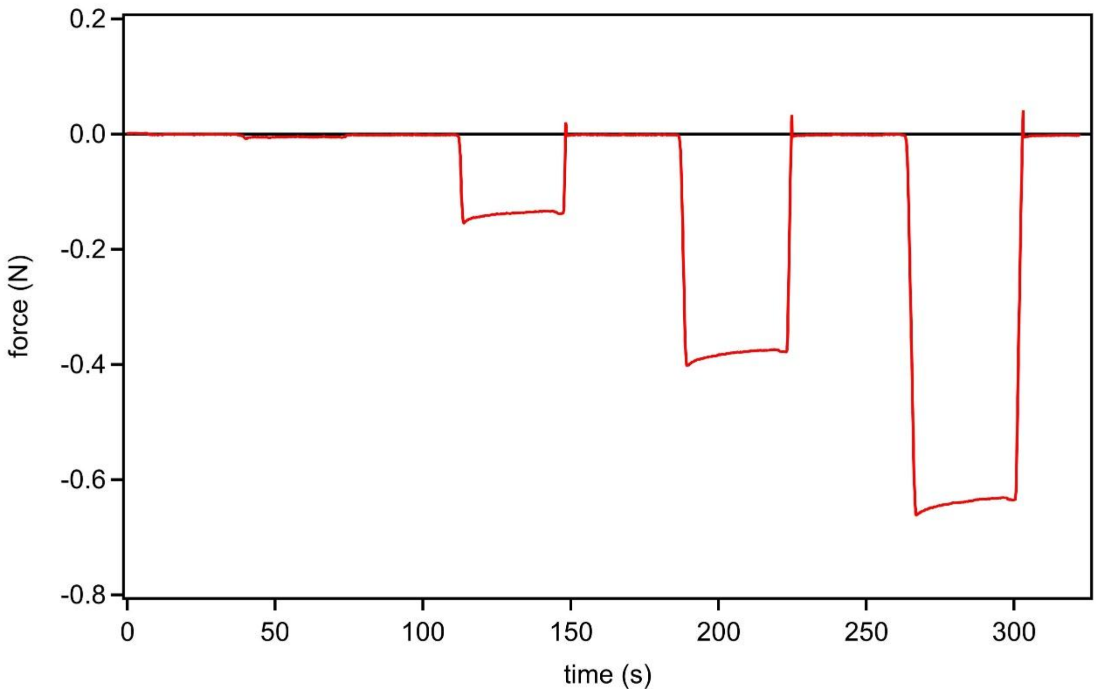

Figure S6. Measured force versus time for two opposing **M75** metasurfaces at room temperature. The adhesive strengths from these measurements reported in Figure 7.

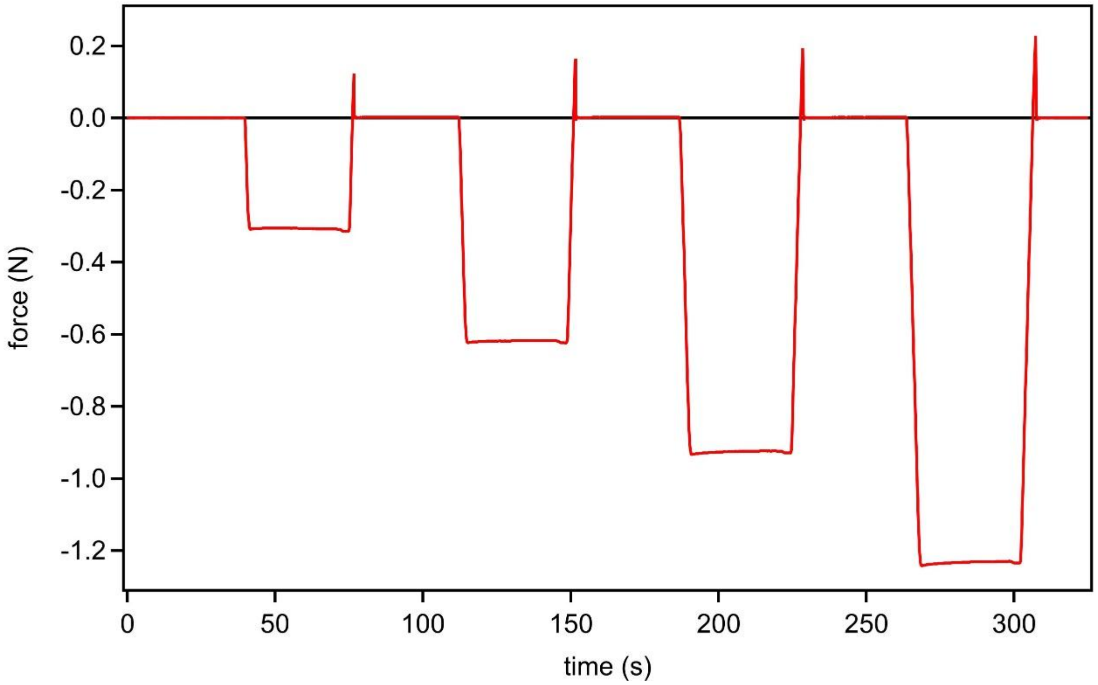

Figure S7. Measured force versus time for two opposing **M75** metasurfaces at room temperature following shape-memory programming (i.e. following the post-shape-memory). The adhesive strengths from these measurements reported in Figure 7.

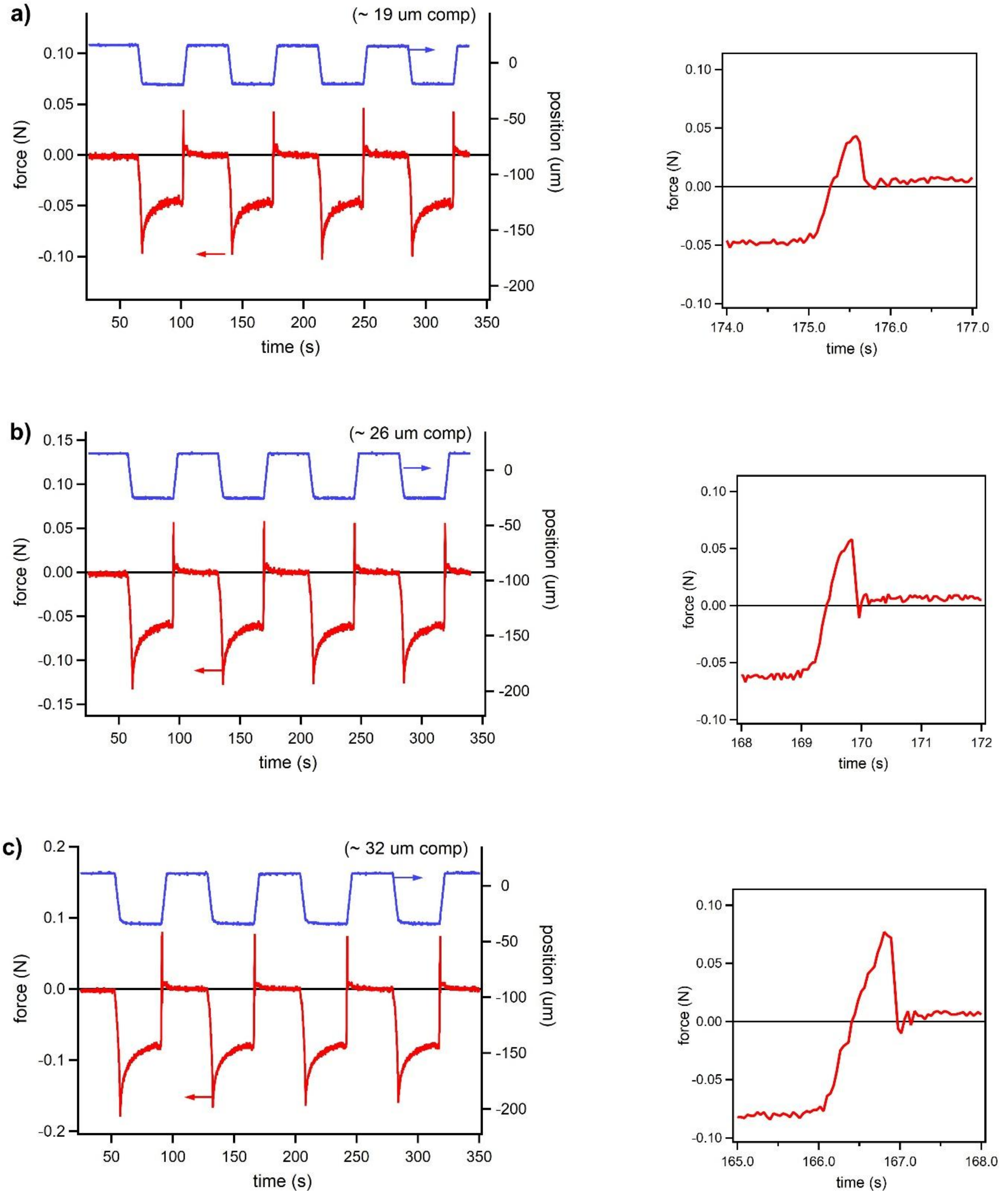

(continued on next page)

Figure S8. Measured force versus time for two opposing **M38** metasurfaces at room temperature at various compressions: (a) 19 μm; (b) 26 μm; (c) 32 μm; (d) 33 μm; (e) 38 μm; (f) 42 μm. The adhesive strengths from these measurements reported in Figure 7.

(continued from previous page)

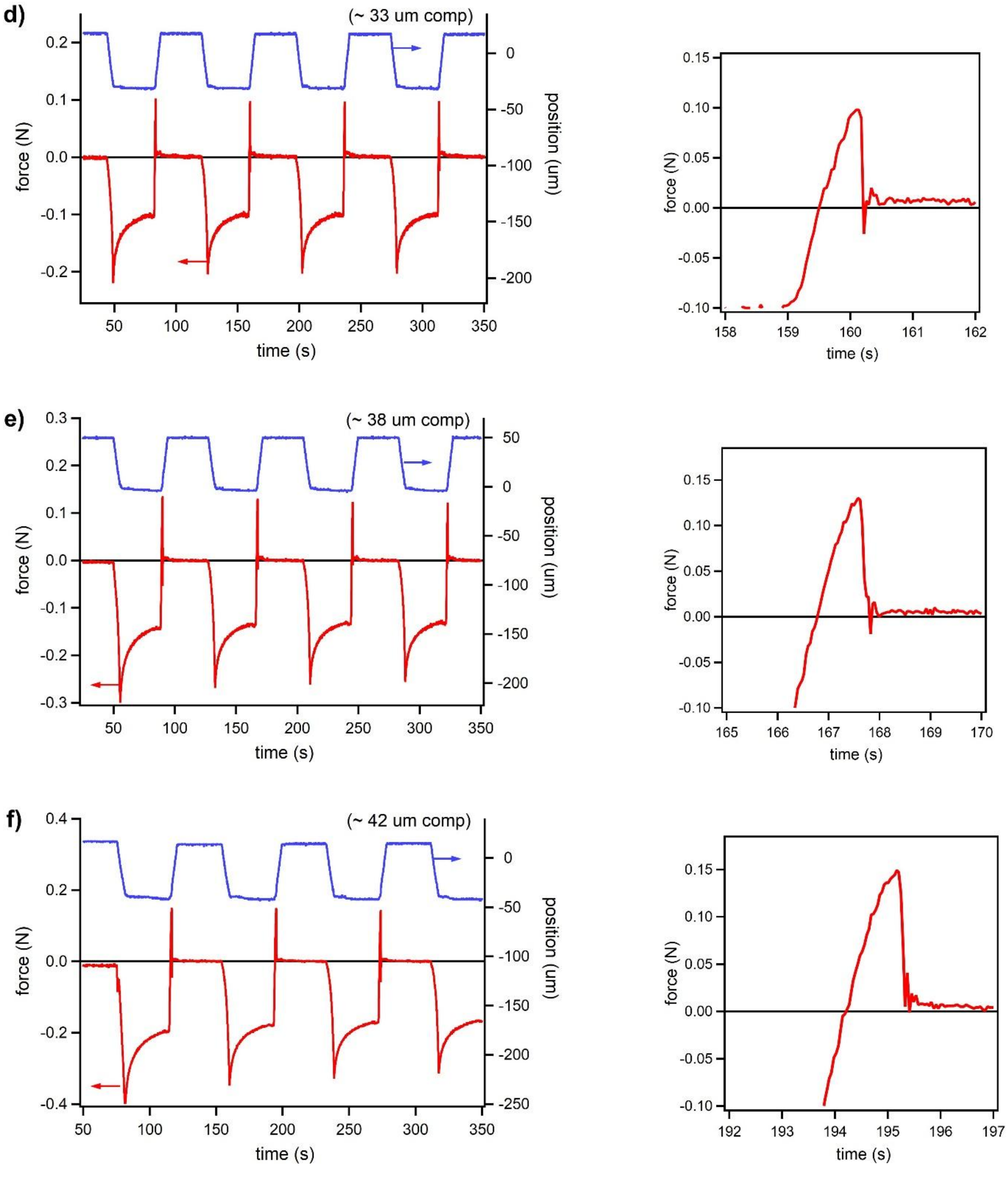

Figure S8. Measured force versus time for two opposing **M38** metasurfaces at room temperature at various compressions: (a) 19 μm; (b) 26 μm; (c) 32 μm; (d) 33 μm; (e) 38 μm; (f) 42 μm. The adhesie strengths from these measurements reported in Figure 7.

The following videos are uploaded as Supporting Information:

Videos are all available at the following static URL:

http://hdl.handle.net/1802/38421

Video S1. Video showing adhesive breaking following compression of a flat print to glass at room temperature.

Video S2. Video showing adhesive breaking of two opposing **M75** prints after compression at room temperature.

Video S3. Video showing adhesive breaking of two opposing **M75**prints after post-shape-memory (PSM) processing.

Video S4. Video showing adhesive breaking of two opposing "chiseled" **M38** prints after at room temperature. The metasurface area of both opposing prints is the center 200 um x 200 um textured region. The surfaces were carefully aligned using a rotation stage and an iterative procedure to ensure the peaks of one print were in registry with the valleys of the opposing print.
